# Melocular Evolution on Cold Temperature Adaptation of Chinese Rhesus Macaques

**DOI:** 10.2174/0113892029301969240708094053

**Published:** 2024-07-10

**Authors:** Xuan Wang, Ming-Hong Feng, Shao-Bo Wang, Hong Shi

**Affiliations:** 1 State Key Laboratory of Primate Biomedical Research, Institute of Primate Translational Medicine, Kunming University of Science and Technology, Kunming,Yunnan, 650500, China;; 2 PET/CT Center, First People’s Hospital of Yunnan Province, Kunming 650032, China

**Keywords:** Melocular evolution, *Macaca fascicularis*, *Macaca mulatta*, temperature adaptation, positive selection, the heat shock proteins

## Abstract

**Introduction:**

Currently, macaques are used as animal models for human disease in biomedical research. There are two macaques species widely used as animal models, *i.e*., cynomolgus macaques and rhesus macaques. These two primates distribute widely, and their natural habitats are different. Cynomolgus macaques distribute in tropical climates, while rhesus macaques mostly distribute in relatively cold environments, and cynomolgus macaques have a common frostbite problem during winter when they are transferred to cold environments.

**Methods:**

In order to explore the molecular mechanisms underlying the temperature adaptation in macaques, genetic analysis and natural selection tests were performed. Based on the analysis of heat shock protein genes, DNAJC22, DNAJC28, and HSF5 showed positive selection signals. To these 3 genes, the significantly differential expression had been confirmed between cynomolgus macaques and Chinese rhesus macaques.

**Results:**

Molecular evolution analysis showed that mutations of DNAJC22, DNAJC28, and HSF5 in Chinese rhesus macaques could enable them to gain the ability to rapidly regulate body temperature. The heat shock proteins provided an important function for Chinese rhesus macaques, allowing them to adapt to a wide range of temperatures and spread widely. The selection time that was estimated suggested that the cold adaptation of Chinese rhesus macaques coincided with the time that the modern human populations migrated northward from tropic regions to relatively cold regions, and the selection genes were similar.

**Conclusion:**

This study elucidated the evolutionary history of cynomolgus macaques and rhesus macaques from molecular adaptation. Furthermore, it provided an evolutionary perspective to reveal the different distribution and adaptation of macaques. Cynomolgus macaques is an ideal biomedical animal model to mimic human natural frostbite.

## 1 INTRODUCTION

Biomedical research is widely used for the non-human primates(NHPs) as model organisms nowadays. Cynomolgus macaques(*Macaca fascicularis*) and rhesus macaques (*Macaca mulatta*) have received a lot of attention from researchers. *M. fascicularis* and *M. mulatta* belong to the genus of macaques, and they are also the most successful and widespread NHPs [1]. Cynomolgus macaques are widely distributed in most parts of Southeast Asia, Malaysia, the Philippines, and parts of the Indonesian archipelago [2-4], they survive in habitats such as tropical monsoon or tropical rainforest climates, and they are located in low-altitude areas. The living environmental temperature of cynomolgus macaques is always above 14.6°C [5]. Rhesus macaques are widespread in mainland China, and northern India [4]. The living environmental temperature of rhesus macaques reaches -7.6°C [5]. From the perspective of the genome, the differences between these two species are very small, and the genetic relationship is very close [6]. The sequence difference between cynomolgus macaques and Chinese rhesus macaques is only 0.34% [7]. However, there are significant differences in morphology, such as weight, height, feeding habits, and distribution areas between cynomolgus macaques compared to rhesus macaques. The cynomolgus macaques are always smaller than rhesus macaques(average weight: rhesus macaques male 9.36 kg and female 6.55 kg; cynomolgus macaques male 5.69 kg and female 3.82 kg) [5].

One of the most obvious phenotypic differences is their adaptation to temperature that cynomolgus macaques are distributed in the subtropical regions, while rhesus macaques spread widely in mainland China, and India. Under cool conditions (13-24°C), cynomolgus macaques increase inter-individual body contact to lower the amount of heat lost by radiation [8], which indicates cynomolgus macaques have a reduced ability to adjust their body temperature in cold environments compared to rhesus macaques. Cynomolgus macaques have a common frostbite problem during the winter when they are transferred to the cool habitats like rhesus macaques. The solution to the frostbite problem always requires manual heating of the breeding environment. Temperature adaptation is an important aspect of the evolutionary process of biological adaptation. The environmental stresses, along with random mutations of primates, shaped the distribution and evolution of primates [9-11]. Rhesus macaques are widely distributed from tropical to northern China, and have adapted to the cool environments. The selection genes that provided the cold temperature adaptation to rhesus macaques are still unknown.

Based on an analysis of the genomic and transcriptomic data, the candidate genes that had underlined natural selection were detected, and the divergence time was estimated. Furthermore, combined with an analysis of the genetic changes to the adaptation of cold temperature in Chinese rhesus macaques, this study would provide genetic data to shed light on the distribution and evolution of macaques. As primates, modern humans are the most widely distributed, and temperature adaptation is also a common feature in different human populations [12, 13]. According to the gene function of humans, these selected genes in macaques and their function were analyzed. In addition, it could provide genetic data and a biomedical animal model to study on human frostbite.

## 2 MATERIALS AND METHODS

### 2.1 Public Genomic Data Achieved

Genome, coding DNA sequence (CDS), and protein sequences were downloaded from the National Center for Biotechnology Information (NCBI) and the National Genomics Data Center (NGDC), respectively. The genome data of cynomolgus macaques is Macaca_fascicularis_5.0 (https://www.ncbi.nlm.nih.gov/genome/776). The genome data of Indian rhesus macaque is Mmul_10 (https://www.ncbi.nlm.nih.gov/genome/215). The genome data of Chinese rhesus macaque is rheMacS (https://ngdc.cncb.ac.cn/gwh /Assembly/653/show) [14].

### 2.2 Natural Selection Test and Divergence Time Estimation

In order to detect genes with positive selection between cynomolgus macaques and rhesus macaque, Ka/Ks analysis was used on all homogenes. The orthogonal group was constructed by orthoFinder [15]. Based on homogenes, paraAT [16] and KaKs_Calculator 2.0 [17], Ka/Ks analysis was performed. The time divergence of each gene was estimated using a synonymous mutation rate of λ substitutions per synonymous site per year, as T = Ks/2λ(λ = 4.27 × 10^-10^) [18]. The divergence time was used to estimate whether the positive selection began after the separation of cynomolgus macaques and rhesus macaques (Table **1**). The sequences of 3 species of macaques were aligned with each other, and JASPAR [19] was used to predict the transcription factor binding site(TFBS).

The genes with positive selection found in macaques, were further explored in human populations because modern human populations are the best sample type for cold temperatures. VCFtools [20] was used to calculate the fixation index (Fst) to estimate the differentiation between the subpopulations in the 1000 genome project phase 3 [21, 22]. The Fst value can also be considered as a measure of positive selection [23, 24]. When the Fst score is greater than 0.15, it could be considered a significant differentiating population. The Fst scores of Eastern Asia(EAS) and the other four populations(African (AFR), American(AMR), European(EUR), and South Asian(SAS)) have been calculated. Finally, three positive selection SNPs of DNAJC22 were used for HUMAN GENOME DATING(https://human.genome.dating/) [25] to infer the time to the most recent common ancestor(TMRCA) and PGG.SNV [26] was used to show the distribution of three positive selection SNPs in the worldwide human populations.

### 2.3 Gene Expression Profiling Analysis

In order to detect whether the positive genes had different expressions between cynomolgus macaques and rhesus macaques, gene expression profiling analysis was executed. RNA-Seq sequencing data came from CRA002684 [27] and CRP000333 [28]. The fastq files for paired-end sequencing of muscle tissue in the two data sets were selected. The sequencing quality of the off-machine data was checked by MultiQC [29], and fastp [30] was used to remove the adapter and low-quality base. Furthermore, hisat2 [31] was used to compare the quality-controlled offline data to the macaque genome (Mmul_10), and Samtools [32] was used to convert the sam file format to bam file format. The original matrix was output by featureCounts [33], DESeq2 [34] was used to analyze differentially expressed genes(DEGs), and ComplexHeatmap [35] was used to display the first 500 DEGs. WGCNA [36] was used for weighted gene co-expression network analysis. Additionally, clusterProfiler [37] was used to enrich the difference gene (Benjamini-Hochberg adjusted *P* value(adjusted-*P* value) of less than 0.05 and absolute Log2(fold change) of more than 1) for GO and KEGG analysis.

### 2.4 Test of Candidate Genes Expression and Genotype

The response of mammals to temperature is not tissue-specific because tissues such as muscles, skin, and blood can quickly respond to temperature changes. The venous blood of three males and females, respectively, in cynomolgus macaques and Chinese rhesus macaques, have been drawn. Total RNA was extracted from blood using an RNA Kit(Omega Bio-tek), and expression of the candidate target gene was tested. EasyScript^®^ One-Step gDNA Removal and cDNA Synthesis SuperMix(Bio-rad) was used for RNA reverse transcription. SYBR™ Select Master Mix (Applied Biosystems) was used for qRT-PCR. The primers were ordered from TsingKe Biotech(Kunming, China) (Table **2**). The internal reference gene is GAPDH [38].

## 3 RESULTS

### 3.1 Positive Selection Genes in Macaque and Human Populations

The darwinian positive selection was detected between cynomolgus macaques with Indian and Chinese rhesus macaques, and the orthogonal groups of these three species were constructed, respectively. Twelve thousand five hundred and ninety-eight and thirteen thousand three hundred and eighty orthologous genes were found in cynomolgus macaques with Indian rhesus macaque, cynomolgus macaques with Chinese rhesus macaque respectively. After Ka/Ks analysis, there were 1410 and 1203 Darwin positive selection genes in Indian rhesus macaque and Chinese rhesus macaque (Fig. **1A**). Four hundred twenty-seven genes were detected with positive selection signals in Indian and Chinese rhesus macaques. Specifically, DNAJC22 had different Ka/Ks scores when Indian rhesus macaque was compared with cynomolgus macaques, and Chinese rhesus macaque was compared with cynomolgus macaques (0.001 and 50). The diversity time of positive selection genes is listed in (Table **1**).

For another gene, HSF5, there were only 239 amino acids in Chinese rhesus macaque, while Indian rhesus macaque and cynomolgus macaques have 615 amino acids, and the deletion of 389 amino acids shaped a huge different HSF5 gene in Chinese rhesus macaque (Fig. **1C**). There were 3 genes with the highest Ka/Ks score(DNAJC22, DNAJC28, and HSF5), and they were annotated to belong to HSPs. There were two single nucleotide polymorphisms(SNPs) on the promotor of HSF5 to achieve two new transcription factor bind sites in Chinese macaques: chr16:44008515(A>G) (transcription factor ALX3 bind site), chr16:44007396(T>C) (transcription factor ASCL1 bind site). There were two SNP mutations on DNAJC22 to achieve two new transcription factor bind sites to Chinese macaques: Chr11: 48194153(T>C) (transcription factor ASCL1(var.2) bind site), and chr11:48194820(A>G) (transcription factor ARGFX bind site) (Fig. **1B**).

The alignment result showed that there were huge differences between Chinese rhesus macaques, Indian rhesus macaques, and cynomolgus macaques in these 3 genes. The Fst value was further explored in modern humans from the 1000 Genome Project database (https://www.international genome.org/) to find if these 3 genes also played an important role in cold adaption in modern humans. The significant Fst score had been found in 3 SNPs of DNAJC22: rs7955245, rs11168981, and rs7310682(Fst score: 0.749692, 0.744666 and 0.713146). Nucleotide diversity(π) and TajimaD test were proven to have a positive selection signal (Table **3**). Those 3 SNPs had different frequencies between Africans(AFR) with other populations (Fig. **2A**), and the data of the Genotype-Tissue Expression(GTEx) project [39, 40] showed that the 3 SNP mutations could up-regulate DNAJC22 expression (Fig. **2B**). The TMRCA of rs7955245, rs11168981, and rs7310682 were estimated to be 48,306.6, 46,561.9, and 47,886.9 years old.

### 3.2 RNA-Seq Reanalysis

The size of the paired-end 150bp sequencing data of cynomolgus macaques and Chinese rhesus macaques were 134.3 Gb and 25.7 Gb, respectively. After removing low-quality bases and sequencing adapters, the clean data was aligned with the Mmul_10 reference genome, and the alignment rate was 87.50 - 92.33%. Totally, 4976 differential genes in the muscle tissues between cynomolgus macaques with Chinese macaques were identified, of which 1499 were up-regulated genes(*P*- adjusted value < 0.05 and Log2(fold change) > 1), and there were 1884 down-regulated genes(*P*-adjusted value <0.05 and Log2(fold change) <1). Considering the selected genes combined with the expression levels of RNA-Seq, 583 genes had positive selection signal and expressing differentiation (Fig. **3A**).

The original matrix of RNA-Seq was converted into TPM [41]. The TPM file was used to perform WGCNA (weighted gene co-expression network analysis), and genes in the expression profile were divided into 53 gene modules (Fig. **3B**). Of 1,318 positive selection genes, 105 existed in one module. Multiple genes belong to the HSPs in the module: DNAJA1, DNAJB9, DNAJB14, DNAJC15, DNAJC16, and DNAJC28.

### 3.3 Quantitative Real-time PCR(qRT-PCR) Analysis

Most of the available transcriptome data of Chinese rhesus macaque originate from the embryonic period, there might be a certain difference with the expression of the muscle group of mature individuals. Therefore, a qRT-PCR test was performed on genes that had positive selection signals. The expression levels of DNAJC22, DNAJC28, and HSF5 in Chinese rhesus macaque were significantly higher than in those of cynomolgus macaques (Fig. **3C**).

## 4 DISCUSSION

The various stress conditions faced by primate populations in the wild include food, habitat climate, and geographic environment. These environmental stresses, along with random mutations of primates, shaped the distribution and evolution of primates [9-11]. The complex habitat geographic environment in Asia has shaped in the adaptive evolution of macaques. However, there were rarely relative studies performed on macaques, especially rhesus macaques and cynomolgus macaques, which were the most successful primates with the largest number and widespread habitats. The physiologic study showed that Japanese macaques had excellent metabolic and thermal responses to cold environments, and the thick fur provided some adaptive advantages [42]. Compared to cynomolgus macaques, Japanese macaques have significantly higher heat production in cold environments [43] and the typical frostbite phenotype of cynomolgus macaque is that the tail ends will gradually necrotize due to poor blood circulation. However, the relative study of rhesus macaques is still unavailable. Gene mutations would provide the metabolic and thermal response to cold temperatures with adaptive superiority to rhesus macaques.

Positive selection signals have been found in the genomes of Chinese rhesus macaques with India rhesus macaques and cynomolgus macaques. In Ka/Ks analysis, HSPs: DNAJC22, DNAJC28, and HSF5 had significant Darwinian positive selection signals. DNAJC22 only experienced positive selection in Chinese rhesus macaques because DNAJC22(ENSMMUG00000007548-GWHPAAIE003399) received a 50 Ka/Ks score while DNAJC22 (ENSMFAG00000026632-GWHPAAIE003399) is only 0.001. This result indicated that only Chinese rhesus macaques had experienced positive selection 29,289 years ago. The deletion of 389 amino acids on HSF5 in Chinese rhesus macaques, HSF5 was gene co-expression. The results suggested that macaques could achieve the ability of body temperature regulation and thermal adaptation through positive selection on HSPs, and it was similar to other mammalians.

Mammalian body temperature regulation and thermal adaptation are mainly through a type of molecular chaperone protein, the HSPs, which can help organisms to confront internal and external stress [44]. Studies have shown that the HSPs gene family plays an important role in heat or cold stress tolerance in different species [44-46]. It has been reported that HSPA8, DNAJC27, and DNAJC28 played an important role in the thermoregulatory protective mechanisms of Nellore(*Bos primigenius indicus*) bulls [46].

The expression of these positive selection genes(DNAJC22, DNAJC28 and HSF5) was tested by qRT-PCR in Chinese rhesus macaques and cynomolgus macaques. It was found that the expression of 3 HSPs in Chinese rhesus macaques was significantly higher than that in cynomolgus macaques. Commonly, HSPs always had a higher expression when they responded to heat or cold stress [44-46]. HSPs provide temperature adaptation for mammals, such as Hsp40, as a chaperone protein of Hsp70, which cooperates to regulate heat adaptation mechanisms in mammalian cells. The differential gene expression between Chinese rhesus macaques and cynomolgus macaques indicated that it could increase the temperature adaptive ability of Chinese rhesus macaques.

It was estimated that this difference between western(India) and eastern(China) rhesus macaques occurred approximately 162,000 years ago, and some Chinese rhesus macaque populations flowed westward to India after their divergence [3]. The divergence time of these positive selection genes was only 32,344 - 60,161 years ago. It indicated that Chinese rhesus macaques had experienced a fast evolution to obtain the quick response to environmental temperature and regulated body temperature in the later stage of the last glacial period.

Compared to Chinese rhesus macaques, modern humans are another primate species that is widely distributed. Homo sapiens developed the adaption of low-temperature tolerance during their migration from Africa. Homo sapiens received morphological and physiological characteristics during this colonization, such as midface [47], regulation of adipocyte differentiation, vasoconstriction, nonshivering thermogenesis, and thermoception [48]. The studies found that positive selections could also affect the gene expression in Homo sapiens to winter temperature adaptation [12]. In this study, 3 HSPs(DNAJC22, DNAJC28 and HSF5) have been detected as positive selection signals in Europe (EUR), East Asia(EAS), America(AMR), and South Asia(SAS) from 1000 Genome Project database. Especially in EAS, 3 most significant SNPs were detected in DNAJC22: rs7955245, rs11168981, and rs7310682, are the Fst scores reached 0.749692, 0.744666, and 0.713146. Nucleotide diversity and TajimaD results showed that EAS experienced positive selection and population extension. Those 3 SNPs were annotated in Ensembl to affect DNAJC22 mRNA expression significantly through changing CTCF binding site (rs7310682) or transfer factor binding site(TFBS) in 3’ UTR variants(rs7955245 and rs11168981) [19].

East Asian ancestors migrated north to the cold environments about 25,000–66,000 years ago [49]. Furthermore, modern humans arrived in Europe about 45,000 years ago [50]. The data shows that the distribution of the three positive selection SNPs in DNAJC22 in the worldwide populations supported the ethnic preference contribution to frostbite. The TMRC times of the three significantly selected loci in DNAJC22 were 46,561.9-48,306.6 years ago, and it coincided with the time that the modern human populations migrated northward from tropic regions to relatively cold regions.

One of the main causes of frostbite is exposure to cold environments, for example, in the Ladakh region at an altitude of 3,650 meters in India, researchers surveyed 1,876 people who suffered from frostbite. There were 108 patients, compared with only 1 patient in the local population. This indicated that the cold adaptation of people at high altitudes might be their greater protection from frostbite [51, 52]. Another study revealed the racial preferences for frostbite, for example, African Americans in the British military have a 30-fold higher risk of injury following cold weather than Pacific Islanders [51]. It suggested that modern humans and macaques could achieve the ability of body temperature regulation and thermal adaptation through positive selection on HSPs, and in this course, mutations that could up-regulate the gene expression were selected out.

This study could improve our understanding of the relationship between eco-geographical changes and inter-species evolution in human and non-human primates. The evolutionary mechanism and diffusion of macaques have always been the focus of genetics and ecology studies. This result suggested that events caused by climate change had a greater impact on the distribution of macaques in subtropical regions than direct inter-specific competition [53]. At the same time, the adaptive evolution of macaques to temperature further linked climate change with genetic mutations indicating that the evolutionary factors of primates could be the result of a combination of multiple factors rather than a single factor [54, 55]. The occurrence of this genetic mutation allowed Chinese rhesus macaques to survive in cold wild areas. Finally, molecular evidence also exists in *Homo sapiens*. In addition, HSPs offered the cold adaptations to EAS. This study also provided a new aspect to the study of cold environmental adaption in rhesus macaques.

The Results revealed that the adaptation to the cold environment played an important role in the widespread distribution of Chinese rhesus macaques. DNAJC22, DNAJC28, and HSF5 in Chinese rhesus macaques could gain the ability to rapidly regulate body temperature and habits. Furthermore, strong positive selections were also found on DNAJC22 in EAS populations that had successfully adapted to cold temperatures. In modern human populations, individuals with ancient alleles of DNAJC22 had a lower ability to adjust their body temperature.

## CONCLUSION

The molecular evolution analysis showed that mutations of DNAJC22, DNAJC28, and HSF5 in Chinese rhesus macaques could enable them to gain the ability to rapidly regulate body temperature, and the heat shock proteins provided an important function for Chinese rhesus macaques to adapt to a wide range of temperature and spread widely. The selection time estimate suggested that the cold adaptation of Chinese rhesus macaques coincided with the time that the modern human populations migrated northward from tropic regions to relatively cold regions, and the selection genes were similar. This study elucidated the evolutionary history of cynomolgus macaques and rhesus macaques from molecular adaptation. Furthermore, it provided an evolutionary perspective to reveal the different distribution and adaptation of macaques. Cynomolgus macaques is an ideal biomedical animal model to mimic human natural frostbite.

## AUTHORS’ CONTRIBUTIONS

X. Wang, S.-b. Wang, and H. Shi conceptualized the project and wrote the first draft of the manuscript. X. Wang and M.-H. Feng contributed to the processing and analysis of the data. X. Wang contributed to the preparation of RNA extraction and qRT-PCR. X. Wang, H. Shi, and S.-b. Wang contributed to guide the data analysis and manuscript writing. All authors contributed to the article and approved the submitted version.

## Figures and Tables

**Fig. (1A):**
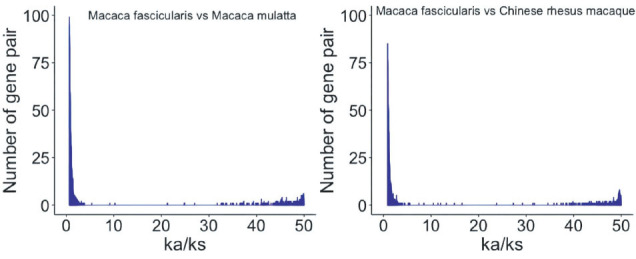
Ka/Ks value distribution in *M. mulatta* and *M. fascicularis*.

**Fig. (1B):**
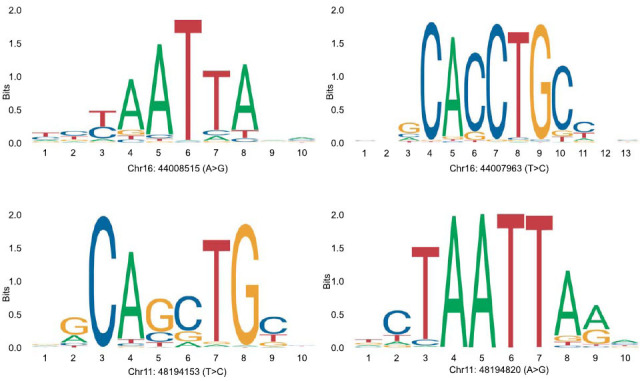
Transcription factor binds predict for *M. mulatta* and *M. fascicularis* in DNAJC22 and HSF5.

**Fig. (1C):**
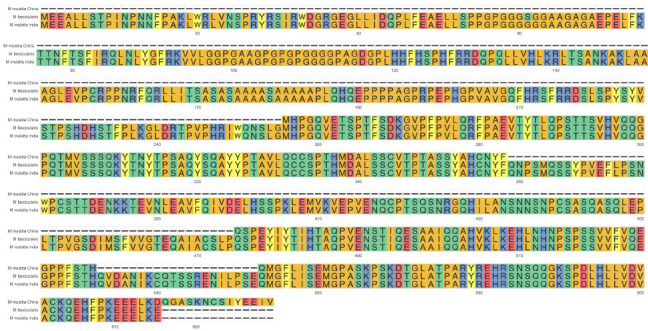
Alignment result of HSF5 in three species: *M. fascicularis*, *M. mulatta* (India) and *M. mulatta* (China).

**Fig. (2A):**
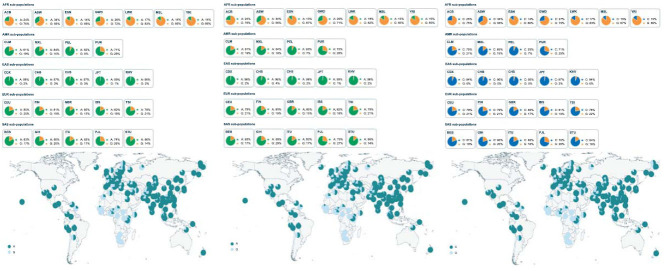
Allele frequency of rs7955245 in the world with Database of PGG.SNV browser.

**Fig. (2B):**
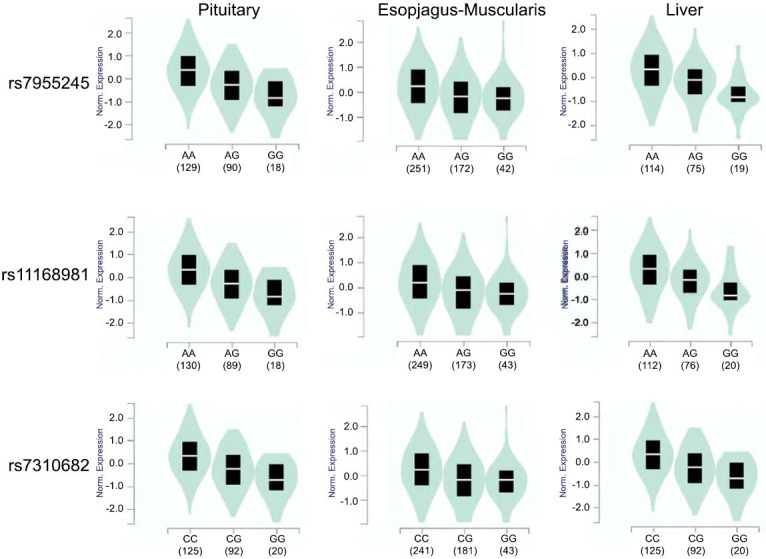
The relationship of 3 SNPs (rs7955245, rs11168981 and rs7310682) and DNAJC22 mRNA expression.

**Fig. (3A):**
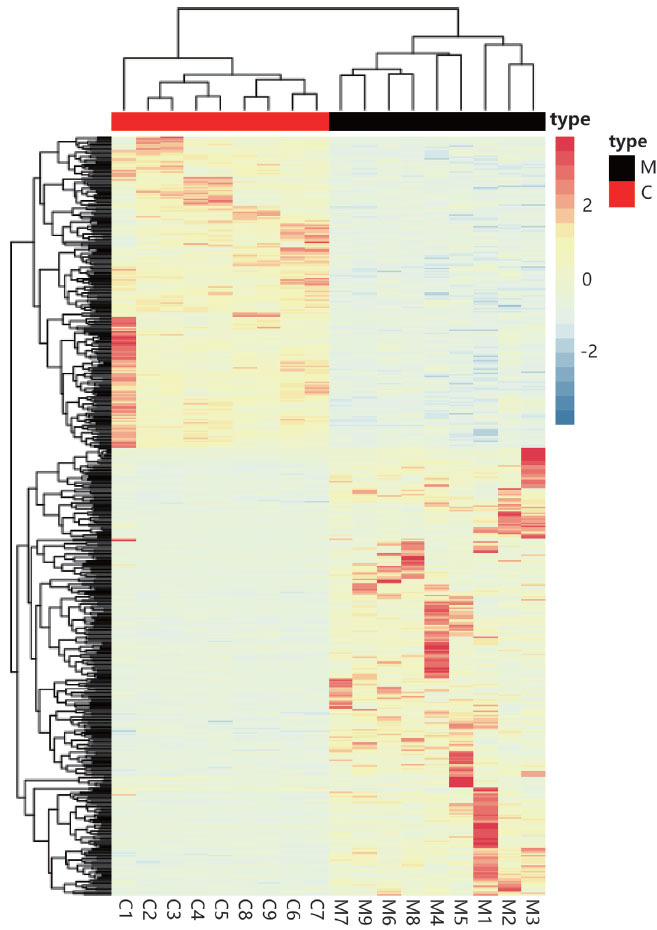
Differentially Expressed Genes(DEGs) in cynomolgus macaques and Chinese rhesus macaques.

**Fig. (3B):**
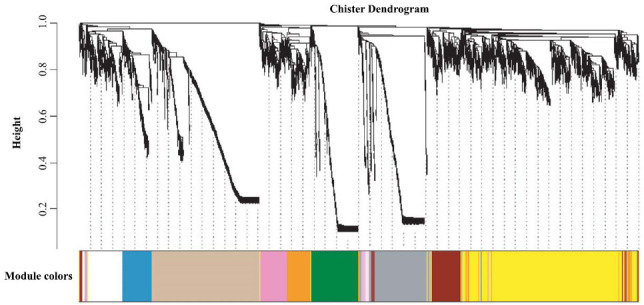
Fifty-three gene modules in Weighted Correlation Network Analysis(WGCNA).

**Fig. (3C):**
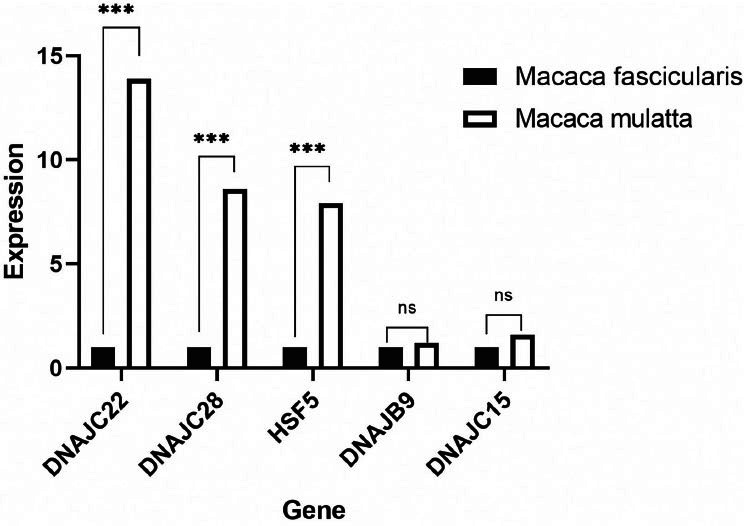
qRT-PCR result of cynomolgus macaques and Chinese rhesus macaques.

**Table 1:** Estimation of positive selection genes’ diversity time in *M. fascicularis* and *M. mulatta*. The beginning character of Genes with ENSMFAG belongs to *M. fascicularis.* The beginning character of Genes with GWHPAAIE belongs to *M. mulatta* (China), and the beginning character of Genes with ENSMMUG belongs to *M. mulatta* (India).

**Gene**	**Orthologs**	**Ks**	**Ka/Ks**	**Diversity Time**
FNDC5	ENSMMUG00000062568-GWHPAAIE000431 (MmI *vs* MmC)	9.35E-05	50	109,515
FNDC5	ENSMFAG00000033251-GWHPAAIE000431 (Mf *vs* MmC)	0.0270697	0.114465	31,697,540
FITM2	ENSMMUG00000029871-GWHPAAIE002228 (MmI *vs* MmC)	3.29E-05	50	38,489
FITM2	ENSMFAG00000044491-GWHPAAIE002228 (Mf *vs* MmC)	0.0509191	0.375683	59,624,238
ACSL1	ENSMMUG00000012177-GWHPAAIE015471 (MmI *vs* MmC)	2.28E-05	50	26,700
ACSL1	ENSMFAG00000033716-GWHPAAIE015471 (Mf *vs* MmC)	0.00530055	0.249817	6,206,733
DNAJC22	ENSMMUG00000007548-GWHPAAIE003399 (MmI *vs* MmC)	2.50E-05	50	29,289
DNAJC22	ENSMFAG00000026632-GWHPAAIE003399 (Mf *vs* MmC)	0.00456024	0.001	5,339,859
HSPB6	ENSMMUG00000002389-GWHPAAIE009907 (MmI *vs* MmC)	4.99E-05	50	58,445
DNAJB7	ENSMMUG00000032244-GWHPAAIE000182 (MmI *vs* MmC)	2.89E-05	45.29	33,823
HSF5	ENSMMUG00000004184-GWHPAAIE007995 (MmI *vs* MmC)	3.7869e-05	50	44,343
DNAJC28	ENSMFAG00000030067-GWHPAAIE012564 (Mf *vs* MmC)	NA	NA	NA

**Note:** Mmc means *M. mulatta* (China); MmI means *M. mulatta* (India) and Mf means *M. fascicularis*.

**Table 2:** qRT-PCR primers for positive selection genes.

**Primer Name**	**Sequence**
HSCB-F	GCAACCGTTCCTTCAGAGTTG
HSCB-R	AATCTGGGTGGACAAGACGC
DNAJC28-F	AGTTCCAGTGACACTTAACATGG
DNAJC28-R	CGGTGTCTCACTCACACATCA
HSF5-F	CCAGCTGGAGCCACTTACTC
HSF5-R	GACTGTGGTAGAGAGCAGGC
DNAJC22-F	GCTTGCAGGGAACATGAGAAA
DNAJC22-R	TGTCAAGAATGCAATAGCCAAGA
HSPA14-F	CGGCACATTCTGTATTGGGC
HSPA14-R	TGCAGACGGTTCGTGGATTA

**Table 3:** Positive selection signals of three SNPs (rs7955245, rs11168981, and rs7310682) in East Asian, European and African populations.

**SNPs**	**Population**	**Nucleotide Diversity**	**TajimaD**	**Fst**
rs7955245	EAS	0.0408391	-0.5662	0.749692
	AFR	0.323498	1.19805	
rs11168981	EAS	0.0446379	-0.54298	0.744666
	AFR	0.326188	1.2146	
rs7310682	EAS	0.0961661	-0.22801	0.713146
	AFR	0.322596	1.1925	

## Data Availability

The authors confirm that the data supporting the findings of this research are available within the article.

## LIST OF ABBREVIATIONS

DEGs: Differentially Expressed Genes
GTEx: Genotype-Tissue Expression
NCBI: National Center for Biotechnology Information
NGDC: National Genomics Data Center
NHPs: Non-human Primates
SNPs: Single Nucleotide Polymorphisms
TFBS: Transcription Factor Binding Site
TMRCA: The Most Recent Common Ancestor
